# Immunological Considerations of Polysorbate as an Excipient in Botulinum Neurotoxin Type A Formulations: A Narrative Review

**DOI:** 10.3390/toxins17120598

**Published:** 2025-12-15

**Authors:** Michael Uwe Martin, Jürgen Frevert, Je-Young Park, Haiyan Cui, Andy Curry, Wei Qi Loh

**Affiliations:** 1 Independent Researcher, 31832 Springe, Germany; 2 Merz Pharmaceuticals GmbH, 60318 Frankfurt am Main, Germany; 3 Apkoo-Jung Oracle Dermatology Clinic, Seoul 06022, Republic of Korea; 4 Department of Plastic and Cosmetic Surgery, Tongji Hospital, School of Medicine, Tongji University, Shanghai 200092, China; 5 Institute of Aesthetic Plastic Surgery and Medicine, Tongji University, Shanghai 200092, China; 6 Merz Aesthetics, Raleigh, NC 27615, USA; andy.curry@merz.com; 7 Merz Asia Pacific Pte. Ltd., Singapore 138589, Singapore; weiqi.loh@merz.sg

**Keywords:** botulinum neurotoxin type A, polysorbate excipients, immunological reactions, hypersensitivity, immunogenicity, risk mitigation

## Abstract

Recent botulinum neurotoxin type A (BoNT/A) formulations have shifted towards the use of polysorbate 20 (PS20) and polysorbate 80 (PS80) as a non-human-derived excipient to enhance product stability. Polysorbates are a distinct class of synthetic non-ionic surfactants with high heterogeneity in chemical structure and properties. Accumulating mechanistic and clinical evidence suggests that they may trigger immunological reactions, including hypersensitivity and immunogenicity. Such risks are largely associated with their susceptibility to degradation via hydrolysis and oxidation, forming reactive byproducts that can interact with proteins and immune pathways. Despite these mechanistic insights, data on the association between polysorbate excipients and observed immune outcomes in practice is relatively sparse and excipient-related immunogenicity and hypersensitivity is often underrecognized in practice. This review provides a summary of polysorbate excipients in BoNT/A formulations, focusing on their chemical properties and degradation pathways, characterizing downstream immune effects and appraising available clinical data of polysorbate-containing BoNT/A formulations. Finally, we discuss potential risk mitigation strategies including process modifications that could prevent degradation, and consideration of alternative excipients, such as human serum albumin, that has been shown to be immunologically inert and has an established safety profile. By integrating chemical, mechanistic, and clinical perspectives, this review seeks to clarify the implications of polysorbate use in BoNT/A formulations and inform both clinical practice and future formulation strategies.

## 1. Introduction

Botulinum neurotoxin type A (BoNT/A) is used to inhibit the release of neurotransmitters at nerve terminals for both therapeutic and aesthetic indications. Early formulations have used human serum albumin (HSA) as a stabilizing excipient due to its proven safety profile over decades [1]. However, difficulties in supply and ensuring the consistent quality of HSA, as well as concerns over potential transmission risks of infectious agents, have led to newer BoNT/A formulations adopting the use of non-human-derived excipients such as polysorbates [2].

Polysorbates are widely used non-ionic surfactants in the pharmaceutical industry due to their ability to act as a detergent, preventing protein adsorption and product loss at product-contacting surfaces such as filters, tubing, primary container/closures and preventing protein degradation at air/liquid interfaces. Moreover, they are thought to stabilize protein structures and reduce aggregation [3], leading to their incorporation as excipients in common biologic drugs and vaccines. While the use of polysorbates in drug formulations have become more common over the years, concerns have emerged regarding their association with detrimental effects, in part driven by their degradation products [4]. These associated effects include immunological reactions (e.g., hypersensitivity), transient injection/infusion-site adverse events (e.g., pain, erythema), and in rare cases renal and hepatic toxicity [4].

Among these, immunological reactions pose an important concern with regard to BoNT/A use for several reasons. BoNT/A is distinctive among biologics as it finds dual utility in both therapeutic and aesthetic indications; increased lifetime exposure and potential overlap between indications can exacerbate risk [5]. These concerns are further underscored by the growing use of intradermal BoNT/A (mesotoxin) in aesthetic practice for skin quality enhancement. Notably, dermal injections carry a higher likelihood of immune activation due to the dense population of antigen-presenting dendritic cells in the dermis [6].

To address these concerns, this narrative review seeks to summarize and examine the implications of the growing use of polysorbate as excipients in BoNT/A formulations, focusing on the chemical properties of polysorbates, downstream immune mechanistic pathways involved, clinical data available on these formulations, and potential risk mitigation strategies.

Our review synthesizes the available literature, highlighting key trends and clinical observations. A systematic review was not performed due to the sparsity and heterogeneity of the evidence. Rather, our goal is to clarify the current landscape and emphasize the need for more standardized practices in future research. To our knowledge, while numerous published reviews have addressed the risks associated with using polysorbate as excipients, no prior reviews have focused specifically on their applications in BoNT/A formulations.

## 2. Chemical Nature and Degradation of Polysorbates

Polysorbates are sorbitan esters of fatty acids, ethoxylated with polyoxyethylene chains: PS20 is primarily composed of lauric acid esters, while PS80 has a high proportion of oleic acid esters. Commercially available PS20 and PS80 are chemically heterogeneous mixtures with varying fatty acid compositions [7]. Regulatory standards for surfactants in biologics vary by compound and country. Multi-compendial grade PS80 typically requires an oleic acid content of ≥58%, while for PS20, the European Pharmacopoeia specifies 40–60% lauric acid esters [7]. These differences complicate characterization of polysorbates and their behavior in formulations.

**Figure 1:**
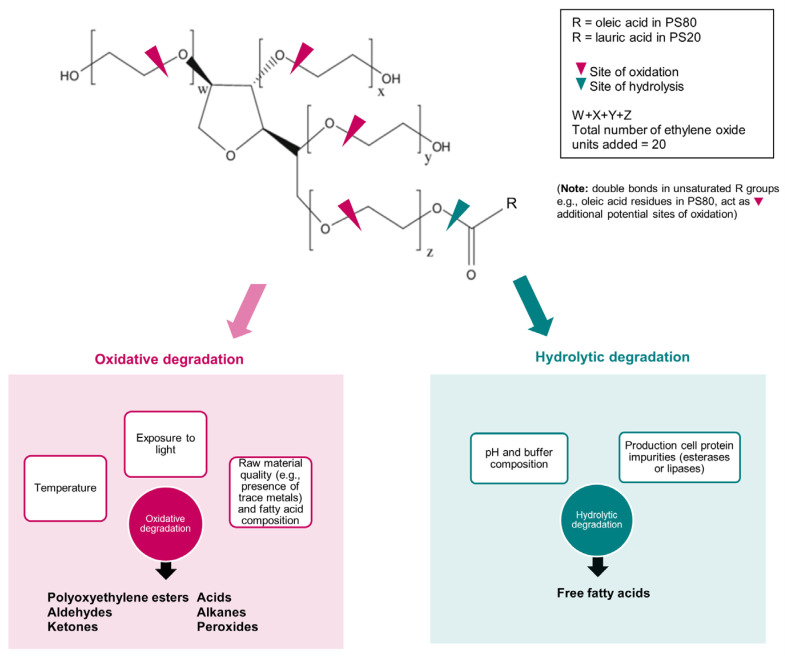
Summary of the oxidative and hydrolytic degradative pathways of polysorbate.

Beyond their chemical heterogeneity, it is well-established that polysorbates as a class of compounds are prone to hydrolysis and autooxidation, forming various degradation byproducts via distinct pathways. These pathways (summarized in Figure 1) are influenced by varying factors, target different reaction sites of polysorbates, resulting in different byproducts [7,8,9,10].

The oxidative degradation of polysorbates is driven by physicochemical conditions and is accelerated at higher temperatures and under greater light exposure [7]. The oxidation of polysorbates is a complex process involving reactions with the polyoxyethelene chains, generating esters, short-chain alkanes, ketones, aldehydes, free fatty acids, peroxides, and acids [10]. The presence of transition metals such as copper or iron also facilitates this autooxidation process [7]. Notably, a stronger degradation rate and earlier oxidation onset was observed for PS80 when compared to PS20 in experimental studies, attributed to its higher oleic acid content and the presence of a double bond that favors hydrogen abstraction [11]. Studies also suggest that more homogenous forms (i.e., all-laureate PS20 and all-oleate PS80) may actually be more susceptible to oxidative degradation [12].

Interestingly, a lower concentration of polysorbate has been associated with higher rates of oxidative degradation [11]. Polysorbate monomers form micelles at higher concentrations, exerting a ‘protective effect’ and making ester bonds less accessible to reactive oxidative substances. This implies that even trace amounts of the surfactant may still contribute to substantial generation of undesired degradation byproducts during storage.

These reactive oxidative byproducts generated can compromise BoNT/A protein stability and increase immunogenicity. Aldehydes, peroxides and ketones can directly oxidize and covalently modify BoNT/A protein, or act as haptens to form neoantigens that trigger immune responses [13,14], and have potential links to toxicological implications [15]. The formation of polyoxyethylene esters and organic acids may promote particle formation by reducing surfactant protection, affecting product quality [7]. Moreover, auto-oxidation [16] results in a self-propagating chain reaction that generates more radicals, leading to accelerated degradation.

On the contrary, hydrolysis mainly involves the cleavage of fatty acid esters (e.g., oleate from PS80 or laurate from PS20) to liberate free fatty acids, and is dependent on pH and the presence of enzymes. The resultant free fatty acids can agglomerate to form subvisible and visible particles, which are known to pose significant challenges to product quality [17,18]. As water serves as the reaction medium for these reactions, liquid ‘ready-to-use’ BoNT/A formulations are considered the most susceptible to the continuous degradation of polysorbate excipients [10].

While chemical hydrolysis involves rapid hydrolysis under acidic or basic conditions, enzymatic hydrolysis arises from the presence of residual production cell proteins with enzymatic capabilities of breaking down ester bonds. The presence of undesired process-related protein impurities is a risk inherent to biologics manufacturing—for BoNT/A, produced from *Clostridium botulinum* bacteria, residual protein impurities from bacterial cultures may inadvertently persist through downstream processing. Notably, studies have identified lipases [19] and novel esterases [20] as part of a complex mixture of proteins secreted from *Clostridium botulinum*. While the proteins produced by widely used mammalian culture systems for the manufacturing of other complex biologics such as Chinese Hamster Ovary (CHO) cells have been extensively studied, in comparison, the literature on residual bacterial proteins from the culture of *Clostridium botulinum* during native BoNT/A production remains limited, underscoring knowledge gaps on a factor that could strongly influence product quality.

Polysorbate degradation byproducts from hydrolysis and oxidative pathways pose significant risks to product quality, leading to various undesired downstream effects [14]. The underlying mechanisms and outcomes are discussed further in the next section.

## 3. Mechanistic Insights and Clinical Implications

**Figure 2:**
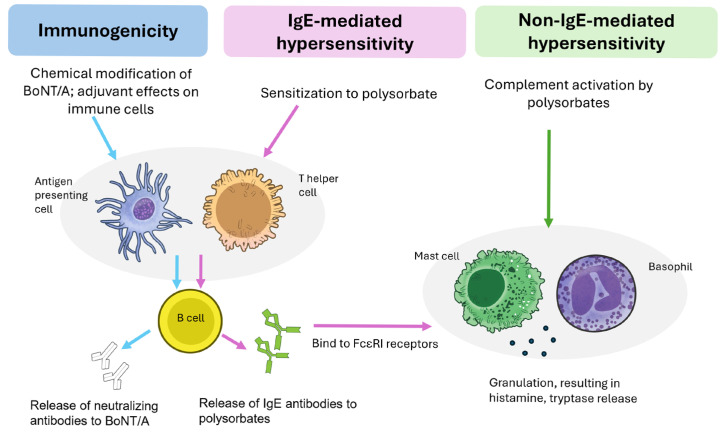
An overview of the distinct immunological pathways elicited by polysorbate. BoNT/A: Botulinum neurotoxin type A; IgE: Immunoglobulin E. The supporting references for the depicted pathways are provided in Section 3.1 and Section 3.2 below.

Here, we discuss the mechanisms and evidence for the broader immunological implications of polysorbate excipient use, with specific reference to the context of BoNT/A formulations. Clinically, such implications may result in diminished efficacy as well as hypersensitivity reactions. The clinical characteristics of such reactions are included in Table 1. A summary of the distinct downstream immunological pathways that lead to these outcomes is provided in Figure 2. 

### 3.1. Risk of Developing Neutralizing Antibodies and Secondary Nonresponse

Immunogenicity is a significant concern with BoNT/A use as the development of neutralizing antibodies (NAbs) renders the neurotoxin inactive, resulting in secondary nonresponse. Once established, all BoNT/A products may exhibit limited effectiveness due to cross-neutralization, severely limiting treatment options for affected patients [5,21].

Reported rates of NAb formation following therapeutic BoNT/A use have varied widely, ranging from 0.3 to 27.6% [5]. For aesthetic applications of BoNT/A, previous reviews have estimated the NAb formation rate to be 0.2% to 0.4%, although true prevalence may be underestimated due to the underreporting of cases in practice [5]. Notably, rates have varied between dose and formulations. A greater prevalence was observed for indications involving larger doses of BoNT/A. When evaluated based on the exclusive use of specific formulations, the frequency of patients with NAbs was the greatest with abobotulinumtoxinA (5.3%), followed by onabotulinumtoxinA (0.6%) and the lowest with incobotulinumtoxinA (0%). In addition, excipients within the BoNT/A formulation may further play a role to modulate immunogenicity [22].

When polysorbate excipients are added to BoNT/A formulations, they can exacerbate immunogenicity by generating reactive degradation byproducts that chemically modify and structurally destabilize the protein [23]. Covalent modifications tend to occur with amino acid residues, especially at exposed nucleophilic sites such as lysine, histidine, and cysteine [14,16]. This can be driven by oxidative degradation products, especially aldehydes, which are extremely reactive electrophiles and can react with primary amino groups of proteins to form adducts with increased immunogenicity [24]. Aldehyde-modified proteins may be recognized as foreign by the immune system, triggering anti-drug antibody responses [25]. In addition, further conformational stress due to surface adsorption and pH changes [16] destabilize the tertiary structure of proteins, which can result in the formation of new antigenic epitopes, or increase their propensity to aggregate. Experimentally, it was shown that light exposure leads to the formation of larger protein aggregates, driven by oxidation of PS80 [26]. Moreover, free fatty acids liberated from the hydrolysis of polysorbates can also organize in droplets to result in proteinaceous aggregates [27,28]. Aggregated or chemically altered BoNT/A protein are recognized more readily by antigen-presenting cells than its native form, raising concerns about long-term exposure to such formulations [29,30,31].

Independently, polysorbates have also been reported to exert adjuvant-like effects. In experimental models, PS80 combined with bacterial antigens elicited a markedly stronger Th2 response—a vital process for antibody production—as compared to antigen alone [32]. PS80 is also an integral component of licensed vaccine adjuvants such as MF59 and AS03; while its primary purpose serves to stabilize emulsion droplets, its structural function indirectly contributes to local inflammation, immune cell recruitment, and enhanced antigen uptake by dendritic cells, all of which are essential for effective adjuvant activity [33]. The ability to form micelles in aqueous solutions has also been attributed to potential depot effects prolonging the uptake of antigens over time [34].

A well-noted real-world example of polysorbate-associated immunogenicity is the surge in incidence of antibody-mediated pure red cell aplasia with subcutaneous epoetin alfa, following the substitution of HSA excipient with PS80 in a formulation [4]. Pure red cell aplasia is characterized by the development of NAbs against erythropoietin, leading to severe anemia, low reticulocyte count, erythroblast absence, and epoetin nonresponse [35]. PS80 was implicated in the increased immunogenicity of the formulation, purportedly due to the formation of epoetin-containing micelles and/or its interaction with leachates with uncoated rubber stoppers of prefilled syringes [36,37]. While the etiology of such phenomena has yet to be fully elucidated, this experience underscores the importance of vigilance to immunological effects when replacing well-established and immunologically inert excipients with polysorbates, as observed for BoNT/A.

### 3.2. Hypersensitivity Reactions

Data on the baseline incidence of hypersensitivity reactions to BoNT/A have been limited [38]. Despite its literature rarity, data from Food and Drug Administration Adverse Event Reporting System (FAERS) from 2014 to 2019 identified allergy as among the top five adverse events, accounting for 3.4% of all adverse events to cosmetic BoNT/A [39]. In an analysis of the EudraVigilance database, anaphylaxis accounted for 0.8% of the total case safety reports reporting BoNT/A as the suspected drug. All cases were reported as serious; 13 cases were reported as ‘life-threatening’ [40]. The spectrum of hypersensitivity reactions can range greatly from mild, transient hypersensitivities to sudden, severe immunologically anaphylactic reactions.

Due to their inherent tendency to induce immune-mediated reactions, the growing use of polysorbates as excipients potentially influence or inflate the risk of hypersensitivity reactions with use of BoNT/A. Literature suggests that the impact of functional excipients on product safety, including anaphylaxis, is often overlooked for biotherapeutics and may be misattributed to the drug itself [14]. The established associations between polysorbates and hypersensitivity reactions based on distinct mechanisms, are summarized below.

#### 3.2.1. Non-IgE Mediated Reactions

Previous studies have identified the associations of PS80 excipients with non-immune anaphylactoid reactions, characterized by histamine release but without any increases in IgE antibodies [9,41]. Although these non-IgE mediated reactions may clinically resemble allergies, their underlying mechanisms are fundamentally distinct and unlike true allergies, these responses do not require any prior sensitization.

In cell culture experiments, PS80 was shown to directly activate mast cells via anaphylatoxins (C3a, C5a and C5b-9) [42]. The upregulation of these complement activation-derived inflammatory mediators supports the hypothesis that exposure to polyethoxylated pharmaceutical detergents like PS80 can trigger acute hypersensitivity such as complement activation-related pseudoallergy (CARPA) [8,43]. While relatively rare, PS80 have also been implicated in delayed cases of organ-specific type IV hypersensitivity to medications following exposure to PS80-containing pharmaceuticals [44].

#### 3.2.2. IgE Mediated Reactions

More recently, the literature suggests that polysorbates may also play a direct role in IgE-mediated reactions [45,46]. In such cases, repeated exposure and subsequent sensitization can lead to the downstream activation of mast cells and basophils as well as histamine release. This is particularly relevant in the context of chronic BoNT/A administration, where cumulative exposure to polysorbate-containing formulations may heighten the risk of sensitization and IgE-mediated hypersensitivity over time.

One additional pressing concern relating to IgE-mediated reactions over time is the potential cross-reactivity between polysorbates and polyethylene glycols (PEGs) due to the structural similarity and common chemical motifs of these compounds [46,47]. With the extensive use of PEGs in pharmaceutical, cosmetic, food, and household products, sensitization to this class of compounds may impose implications on patient safety and quality of life [48], and while relatively rare, could impact future therapeutic options with drugs that are PEGylated [49,50].

### 3.3. Current Clinical Experience with Polysorbate-Containing BoNT/A Formulations

Several polysorbate-containing BoNT/A products have become commercially available, and an overview is provided in Table 2. The majority of these were approved in various markets in the past five years and extensive real-world data on the risk of hypersensitivity or immunogenicity of these formulations is not yet available. Evaluations from clinical trials may serve as a useful reference, although these may not fully represent the immunological risks in practice and over time.

Phase 3 single-dose studies on daxibotulinumtoxinA for the treatment of moderate-to-severe glabellar lines and cervical dystonia with follow-up up to 36 weeks did not report any incident cases of hypersensitivities or immunogenicity [58,59]. However, in the open-label extension study for moderate-to-severe glabellar lines administering three doses over a longer follow up (36–84 weeks) in 2691 patients, one adverse event of hypersensitivity assessed as possibly related to the intervention was reported [60]. In the same study, one subject had tested positive for NAbs at screening and throughout the study, although causality was not concluded as the subject had previously received botulinumtoxinA. Similarly, in a longitudinal open-label study of 357 patients for the treatment of cervical dystonia assessing up to four doses over 52 weeks of follow-up, one patient developed NAbs de novo and sustained positivity throughout follow-up [61]. Although attributed to prior rimabotulinumtoxinB treatment, cross-reactivity across toxin subtypes is thought to be unlikely. Overall, findings suggest that short-term, placebo-controlled studies may demonstrate a low incidence of immunogenicity and hypersensitivity but longer-term data across larger populations will allow a more nuanced understanding of risk.

Pooled analyses of single-dose studies of relabotulinumtoxinA in glabellar lines and/or lateral canthal lines with up to 6 months of follow-up (READY-1, READY-2 and READY-3) detected no NAbs [62]; however, published literature providing granular longitudinal data on immunogenicity and hypersensitivity is not yet available. On the contrary, in one longitudinal study of abobotulinumtoxinA RTU for the treatment of moderate to severe glabellar lines (595 patients treated for up to 5 total doses, with mean follow-up of up to 377.2 days), no NAbs were observed, but hypersensitivity reactions were noted in two patients (0.3%): two events of eye allergy in a single patient and rash in another patient, although these were not confirmed as related to treatment [63,64].

Multiple phase 3 studies have evaluated nivobotulinumtoxinA for the treatment of glabellar lines, lateral canthal lines, or the concurrent treatment of both indications [65,66]. Among the findings, there were indications of treatment-induced NAb development in two individuals out of a total of 556 patients, who received up to three treatment cycles, with follow-up until 360 days [65]. One patient demonstrated onset of NAbs at Day 90 after receiving nivobotulinumtoxinA for both glabellar lines and lateral canthal lines. Another patient tested positive for NAbs after treatment initiation with nivobotulinumtoxinA for glabellar lines, with loss of treatment response at all subsequent visits. Although a pre-treatment result for NAbs was not available, the clinical picture is indicative of NAb-mediated secondary nonresponse.

Meanwhile, published data on Coretox remains limited. One phase 3 study evaluating its use in post-stroke upper limb spasticity was published, and notably this paper did not include any evaluations of immunogenicity [67]. The lack of publicly available data for such evaluations hinders estimates of the incidence of immune-related reactions, or contributions of excipients.

Moreover, for PS20, majority of the safety studies have been performed with non-invasive route of administrations, i.e., oral toxicity and skin irritation, with the only published supportive study on intramuscular administration in the rodent species having limited duration of follow-up of three injections over four weeks [2].

To date, meaningful assessments of the incidence of NAbs and hypersensitivity reactions to polysorbate-containing BoNT/A formulations are constrained to isolated case descriptions, hindered by the short duration of follow-up and limited sample sizes of these studies. Moreover, NAb test methods employed across studies may differ in varying sensitivities, specificities, and cut-off criteria, making comparisons challenging. These uncertainties highlight the need for further clinical and experimental data to clarify the impact of polysorbate in BoNT/A formulations, as well as the importance of ongoing pharmacovigilance and risk mitigation strategies.

## 4. Risk Mitigation

The US Pharmacopeia, US FDA, and Europeans Medicines Agency (EMA) have acknowledged the adverse effects of polysorbate degradation, however there are currently no formal statements or regulations requiring tests for polysorbate degradation [68,69,70]. More recently, EMA proposed that for polysorbate-containing products administered by parenteral routes (e.g., intramuscular, subcutaneous), a warning on allergic reactions should be included in product leaflets considering the risk for hypersensitivity reactions including anaphylactoid shock upon exposure to intact polysorbates entering the bloodstream [71].

Nascent research initiatives have emerged to address the complex risk landscape posed by polysorbate excipients in pharmaceutical formulations. This topic has received increased scrutiny by biotechnology stakeholders, prompting efforts to review industrial practices and identify potential gaps [10,72].

While these measures reflect growing attention within the manufacturing sector, awareness of polysorbate-related risks remain relatively unacknowledged in aesthetic or therapeutic practice. This highlights the importance of greater awareness among clinicians, as more polysorbate-containing BoNT/A formulations become available. Ultimately, effective risk mitigation necessitates efforts from both manufacturers and clinicians, reflecting distinct responsibilities throughout product lifecycles.

### 4.1. Manufacturers

Various strategies could be employed to address polysorbate degradation, depending on whether oxidative or hydrolytic degradation pathways are being targeted.

To mitigate oxidative degradation, manufacturers may source for polysorbates that demonstrate low peroxide generation and sustained quality (e.g., minimal metal impurities). Product selection should consider that certain subspecies composition—such as high proportion of oleate or laureate esters—can lower oxidative stability [12]. Further strategies involve stringent control of manufacturing and storage parameters (low temperature, minimal light, anaerobic environment) while considering the use of radical scavengers, e.g., methionine and cysteine, to reduce radical-mediated oxidation [72]. To minimize interactions with leachable materials, the use of inert, single-use container materials (e.g., disposable polymer bags) as opposed to materials like stainless steel has also been recommended [73]. Perhaps owing to the availability of effective mitigation measures for oxidative degradation, polysorbate oxidation is reportedly not perceived as a frequent problem, as compared to hydrolysis, by industry representatives.

Hydrolysis—particularly enzyme-mediated hydrolytic degradation—has been identified as a much greater concern than oxidation [72]. One major challenge with addressing this phenomenon is the elusive nature of residual production cell proteins—also called host cell proteins in other expression systems (e.g., recombinant mammalian cell lines)—because those with enzymatic activities can break down substrates at high activity even at very low concentrations beyond detection limits. Moreover, production cell lines may differ in the enzyme expression levels and their varied target specificities, leading to distinct degradation patterns [74]. Notably, switching to a lyophilized formulation was considered an effective solution by industrial stakeholders based on the aforementioned industrial survey, as water serves as a vital reaction medium for enzyme-mediated hydrolysis.

Another factor that could be used to control the rate of hydrolysis would be to reduce storage temperature to slow down enzyme kinetics [75], or to replace polysorbate with alternative non-ionic surfactants or stabilizers [76]. Other strategies include the addition of enzyme inhibitor probes to inhibit hydrolytic activity of production cell enzymes [77], or the knockout of genes coding for pertinent enzymes in production cell cultures using technologies such as CRISPR (clustered regularly interspaced short palindromic repeats) [78]. More recently, proactive efforts to monitor activity of polysorbate-degrading enzymes have been developed with the aim of optimizing manufacturing processes [79]. In an industrial survey, six out of 16 companies reported the use of enzymatic activity assays for investigational purposes, particularly in the manufacturing of drug substance for biologic interventions [10]. Yet, such methods may not always be possible or feasible, especially since they require the definitive identification of polysorbate-degrading production cell enzymes, in this case from *Clostridium botulinum* cultures.

Beyond manufacturing processes, post-marketing pharmacovigilance efforts by manufacturers and marketing authorization holders help to provide an evidence-based assessment of the long-term implications of polysorbate excipients in BoNT/A. Such efforts should incorporate comprehensive, periodic assessments to detect associations of any contributing factors with unwanted adverse reactions of hypersensitivity and immunogenicity.

### 4.2. Clinicians

Clinician awareness of the immunological implications of polysorbate excipients is critical for informed decisions, and most pertinent when switching patients from HSA based formulations to polysorbate-containing BoNT A formulations or treating individuals with a history of secondary nonresponse or suspected polysorbate hypersensitivity. In such cases, careful patient selection and monitoring are essential to avoid unintended immunogenic responses and ensure effective management of reactions. Moreover, consideration should also be given to the intended mode of administration, as intradermal applications may come at a higher risk of immunological reactions.

Equally important is clinical adherence to proper storage and handling protocols, as deviations such as temperature excursions or prolonged exposure to light, can accelerate polysorbate degradation and potentially increase the risk of adverse reactions. While appropriate storage is generally expected in clinical settings, variability in practice underscores the need for reinforced compliance and staff training.

Clinicians also play a crucial role in ensuring that any potential immunological reactions toward polysorbate-containing BoNT/A are accurately documented. Notably, variations in safety profile may exist within polysorbate-containing BoNT/A formulations, driven by the specific type of polysorbate used, the concentration of the excipient, and the presence of stabilizers or antioxidants. As such, consistent pharmacovigilance practices enable the industry to capture a more accurate product-specific safety profile, facilitating evidence-based assessments and informing future formulation decisions. Notably, to avoid underreporting, clinicians should be cognizant that immunological reactions may not necessarily be attributed to the toxin itself, but can also arise from reactions to excipients present in the product [13,14]. At the same time, it is acknowledged that efforts to determine causality can often be hindered by the limitations of the types of clinical tests available in regular practice settings.

When evaluating risk, clinicians should also consider the relative safety track record of alternative excipients in BoNT/A formulations. Poloxamer 188, another synthetic non-ionic surfactant, was recently used in place of polysorbate in a newly approved BoNT/A formulation CKDB-501A (Tyemvers, Chong Kun Dang BIO Corporation, Seoul, Republic of Korea), citing concerns over potential polysorbate-associated hypersensitivity and anaphylaxis [80,81]. While poloxamer 188 may offer improved oxidative stability, long-term safety follow-up is limited and experimental data have also demonstrated auto-oxidation (degradation of polyethylene and polypropylene oxide chains) and particle formation after long-term storage [82,83,84].

Meanwhile, the totality of data demonstrates that HSA is a biologically compatible excipient with a strong safety profile based on decades of clinical and supplier safety data at concentrations used in BoNT/A formulations [1]. The safe use of HSA in greater doses has also been demonstrated as a therapeutic—in a meta-analysis of approximately 16.2 million doses (40 g) of HSA administered from 1998 to 2000, only 198 non-fatal serious adverse events and 13 fatal serious adverse events were recorded, corresponding to 4.65 and 0.77 events per million doses, respectively [85]. Of these, only a small fraction (7.1%) were assessed as probably related to HSA; none of the fatalities were deemed likely to be linked to HSA.

Further, immunologically mediated adverse events have not been attributed to HSA a stabilizer in BoNT/A products. Established formulations using HSA therefore offer a lower risk profile considering the robust long-term safety data available. Longitudinal data for polysorbates, or even poloxamer 188, as an excipient for BoNT/A have been limited and true evidence-based assessments will only be possible as post-marketing safety data accumulates over time.

## 5. Conclusions

Polysorbates offer a non-human alternative to HSA for stabilizing BoNT/A while avoiding theoretical pathogen risk, but their use is not without risk. Mechanistic evidence suggests that the degradation products of polysorbates can lead to immunological complications, driven by the reactive products from established oxidative and hydrolytic degradation pathways of polysorbates. Existing literature suggests that polysorbate may account for hypersensitivity reactions or immunogenicity of polysorbate-containing interventions. Further research is needed to better characterize the long-term safety of polysorbate as an excipient for BoNT/A. To minimize risk, manufacturers should adopt a pro-active stance in monitoring product quality and consider adopting appropriate risk mitigation strategies during manufacturing processes. Clinicians should exercise caution given the increased availability of polysorbate-based formulations, especially in immunologically vulnerable or heavily treated populations. Concerted pharmacovigilance efforts will help to facilitate evidence-based assessments and inform future formulation decisions as real-world data accumulates.

## Figures and Tables

**Table 1:** Characteristics of immune downstream effects from polysorbate excipient exposure in BoNT/A formulations.

	Immunogenicity	IgE-Mediated Hypersensitivity	Non-IgE Mediated Hypersensitivity
Clinical presentations	Loss of efficacy over time (secondary nonresponse)	Hypersensitivity symptoms such as itching, swelling, urticaria	
Time to onset	Activation of antigen-presenting cell complex within hours and adaptive immune system over days/weeks. Clinical manifestation of secondary nonresponse may occur months or years later.	Immediate (seconds to a few hours) upon exposure	
Detection methods	Binding antibody assays, e.g., ELISA as a screening test	Skin prick test against polysorbates	Increase in circulating complement mediators, e.g., C3a/C5a without IgE involvement
	Neutralizing antibody tests (mouse hemidiaphragm assay) as a confirmatory test	Serum-specific IgE test against polysorbates	

BoNT/A: botulinum neurotoxin type A; IgE: Immunoglobulin E; ELISA: Enzyme-linked immunosorbent assay.

**Table 2:** BoNT/A formulations containing polysorbate as an excipient.

Product Name	Generic Name or Code Name	Initial Approval (Year)	Form	Polysorbate Excipient Content	Storage Conditions
DAXXIFY^®^ [51]	DaxibotulinumtoxinA	United States (2022)	Lyophilized powder	PS20 (0.1 mg per 50 U or 100 U vial)	At RT, protected from light.
Relfydess^®^ [52]	RelabotulinumtoxinA	Europe (2022)	Liquid, ready-to-use	PS80 (1.6 mg per 150 U vial, or 1.1 mg/mL)	At 2 °C to 8 °C. Unopened vial may be brought to RT at 25 °C while protected from light. Stability for unopened vials has been demonstrated up to 24 h at RT.
Alluzience^®^ [53,54]	AbobotulinumtoxinA RTU	Europe (2021)	Liquid, ready-to-use	PS80 (0.1 mg per 1 mL, 500 U vial)	At 2 °C to 8 °C. May be held at up to maximum of 25 °C for a single period of 12 h when unopened and protected from light.
Coretox^®^ [2,55]	MT10107	South Korea (2016)	Lyophilized powder	PS20 (1.0 mg per 100 U vial)	At 2 °C to 8 °C in hermetic container.
Innotox^®^ [56,57]	NivobotulinumtoxinA	South Korea (2013)	Liquid, ready-to-use	PS20 (0.094 mg per 50 U or 100 U vial)	At 2 °C to 8 °C in hermetic container.

BoNT/A: Botulinum neurotoxin type A; RT: Room temperature; RTU: Ready-to-use.

## Data Availability

No new data were created or analyzed in this study. Data sharing is not applicable to this article.

## Abbreviations

The following abbreviations are used in this manuscript:

BoNT/A	Botulinum neurotoxin type A
CARPA	Complement Activation-Related Pseudoallergy
CHO	Chinese Hamster Ovary
CRISPR	Clustered Regularly Interspaced Short Palindromic Repeats
EMA	European Medicines Agency
ELISA	Enzyme-linked immunosorbent assay
FDA	Food and Drug Administration
HSA	Human serum albumin
IgE	Immunoglobulin E
NAb	Neutralizing antibody
PEG	Polyethylene glycol
PS20	Polysorbate 20
PS80	Polysorbate 80
RT	Room temperature
RTU	Ready-to-use
